# Barking up the Wrong Tree: Why and How We May Need to Revise Alcohol Addiction Therapy

**DOI:** 10.3389/fpsyg.2017.00884

**Published:** 2017-05-29

**Authors:** Ann-Kathrin Stock

**Affiliations:** Cognitive Neurophysiology, Department of Child and Adolescent Psychiatry, Faculty of Medicine, Technische Universität DresdenDresden, Germany

**Keywords:** alcohol, addiction, AUD, control, habit reversal therapy, inhibition, therapy

## Abstract

One of the main characteristics of alcohol abuse and addiction is the loss of control over alcohol intake and the continuation of drinking in the face of negative consequences. Mounting evidence strongly suggests that an alcohol-induced imbalance between goal-directed and habitual behavior may be one of the main driving factors of this key feature of addiction and furthermore play a key role in staying abstinent. Current therapies often focus only on deficient inhibitory control (i.e., goal-directed behavior), but largely neglect the potential of the well-functioning habit formation found in patients. Yet, focusing on intact habitual/automatic mechanisms in addition to or maybe even instead of deficient cognitive control might equip us with a more effective tool to battle the current alcohol abuse and addiction epidemic, especially with respect to more severely impacted patients who likely suffer from permanent alcohol-induced brain damage. Against this background, I would like to advocate the application and scientific evaluation of habit reversal therapy (HRT) for alcohol abuse and addiction.

## Introduction

In many, if not most parts of the world, alcohol abuse and addiction are problems of epidemic proportions which do not only cause a wide range of health problems for the affected individuals, but also skyrocketing costs for healthcare systems as well as a great number of socioeconomic problems (WHO, 2016). Considerable efforts are made to alleviate the adverse effects of this epidemic, but most countries have not had noteworthy decreases in alcohol consumption per capita within the last 25 years^1^. Also, relapse rates among alcohol use disorder (AUD) patients have remained extremely high across different currently used therapeutic approaches, with often more than 50%^2^ of patients consuming again after completing therapy (Garbusow et al., 2014; Naqvi and Morgenstern, 2015). Against this background, we are in dire need of better treatment options. One of the possible reasons for the low success rates of current AUD treatments is that even though the last decades have seen an unprecedented surge in alcohol abuse and addiction research, many clinical therapeutic approaches do not (yet) consider the latest findings. And while this is not the case for evidence-based treatments, it has recently been noted that even those are currently only modestly effective (Naqvi and Morgenstern, 2015).

In order to improve the current situation, effective therapeutic interventions need to be rooted in a mechanistic, not just a correlational, understanding of the behavioral and neurobiological changes that cause harmful consumption and lead to relapse in AUD patients. Based on advances in basic cognitive neuroscience research on the effects of alcohol on the nervous system and behavior, we might now be able to rise to this challenge. In the light of accumulating evidence that alcohol seems to shift the healthy balance between goal-directed and habitual behavior towards the latter, it appears that we might have been barking up the wrong tree all along: Mainly focusing on the cognitive control deficits observed in AUDs, we have utterly neglected cognitive functions such as habits and automatisms, which have lately been proven to remain largely preserved. This is quite unfortunate as preserved cognitive functions provide promising working points to establish alternatives or additions to currently popular therapeutic approaches. As this potential opportunity might benefit millions of patients, current approaches and potential alternatives will be contrasted and discussed in the following.

## Alcohol and Control Deficits

One of the key problems contributing to relapses in AUD patients are executive control deficits which result in the inability to control alcohol intake and lead a productive, self-serving life (Fein and Cardenas, 2015; Koob and Volkow, 2016). The term ‘executive control’ subsumes several cognitive functions that help us to adapt to new situations, solve problems, and, perhaps most importantly, counteract impulsive or automatic behavior (for a detailed review, see Diamond, 2013). Among all executive functions, inhibitory control plays a special role in alcohol abuse (Copersino, 2017). It is defined as our ability to control thoughts, emotions, attention and behavior in order to resist temptations, internal predispositions or habits and replace them with more appropriate, goal-directed behavior (Diamond, 2013). Being able to control habits is key to maintaining abstinence as habitual actions substantially contribute to addiction (McKim et al., 2016). In the early stages of substance (ab)use, the consumption of alcohol is usually motivated by the reinforcing hedonic effects of alcohol, but probably due to an interaction of pavlovian and instrumental learning, repeated self-administration gradually shifts the mechanisms driving behavior to stimulus-response (S-R) associations. This eventually leads to the formation of habits and compulsions which are no longer sensitive to outcome devaluation (Everitt and Robbins, 2005; de Wit and Dickinson, 2009; McKim et al., 2016) because alcohol consumption is no longer driven by expected outcomes, but instead triggered by alcohol-associated stimuli (de Wit and Dickinson, 2009; McKim et al., 2016).^3^ In practical terms, this behavioral autonomy means that AUD patients tend to maintain their alcohol consumption even in the face of negative consequences, which contributes to the development and maintenance of addictive behavior in AUD (Corbit and Janak, 2016; López et al., 2016). Of note, this effect extends to other behavioral domains as well since alcohol has been shown to shift even consumption-unrelated behavior from goal-directed towards habit-based processes (e.g., Stock et al., 2016) and to generally reduce goal-directed executive control capacities including behavioral inhibition (Brion et al., 2014; Garbusow et al., 2014; Day et al., 2015; Fein and Cardenas, 2015; Trantham-Davidson and Chandler, 2015; Koob and Volkow, 2016).^4^

**FIGURE 1:**
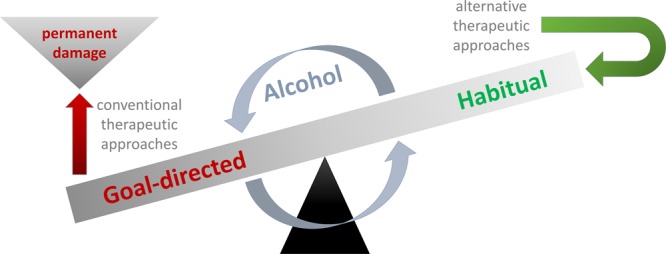
Alcohol impairs the balance between goal-directed and habitual behavior so that habitual behavior like compulsive drinking can no longer be kept in check by goal-directed control mechanisms such as inhibition. Many conventional therapies primarily aim at improving/augmenting goal-directed cognitive control so that habitual drinking can be overcome. Unfortunately, alcohol abuse may permanently damage frontal brain areas and thus diminish control faculties so that quite a few AUD patients may never develop goal-directed behavior that can effectively keep their drinking habits in check. Against this background, I would like to espouse alternative therapeutic approaches like HRT which aim at modifying or changing habits instead of trying to inhibit them via goal-directed behavior (for details, please see Habit-Based Treatment Options).

Altogether, these changes result in a dysfunctional state where behavioral control is reduced, while the automatisms it should keep in check prevail or may even become enhanced over the course of an AUD (see **Figure 1** for illustration). Based on this lack of control capacities, it may seem like the most logical consequence to try to enhance executive functioning/cognitive control in AUD patients (Verdejo-Garcia, 2016), who may present with sometimes severe impairments of this cognitive domain and therefore fail to abstain from drinking (Harper, 2007; Brion et al., 2014). In line with this approach, it has been shown that cognitive control training like goal management training (GMT) may improve executive functions in individuals with substance use disorders (Alfonso et al., 2011). Furthermore, cognitive control training seems to have mildly beneficial effects on the alcohol consumption of non-addicted heavy drinkers (Berg, 1948) and individuals with hazardous drinking behavior who reported relatively strong automatic preferences for alcohol (Houben et al., 2011). Yet still, it is questionable whether more severely impaired AUD patients who already suffer from alcohol-related brain damage and/or Korsakoff’s syndrome (KS) are able to benefit from cognitive control training. The reason for this assumption is that severe alcohol abuse and the resulting thiamine deficiency often lead to brain damage including thalamic or frontal cortical atrophy (Brun and Andersson, 2001; Oscar-Berman et al., 2004; Matsumoto, 2009; Oscar-Berman, 2012; Maharasingam et al., 2013; Pitel et al., 2015) as well as functional changes within fronto-striatal loops and the dopaminergic and GABAergic transmitter systems (Everitt and Robbins, 2005; Gremel and Costa, 2013; Sjoerds et al., 2013; Barker et al., 2015; Koob and Volkow, 2016; Gremel and Lovinger, 2017), all of which may which cause severe executive control deficits that do not necessarily seem to fully to recover with abstinence (Thomson, 2000; Harper, 2007; Trantham-Davidson and Chandler, 2015). And as consciously controlling habitual drinking heavily strains cognitive control capacities, this means that cognitive control training and related standard addiction treatments may only benefit patients in early stages of AUD who have not yet suffered substantial damage to the brain areas mediating this cognitive faculty (Copersino, 2017; Gladwin et al., 2017).

## Habit-Based Treatment Options

At this point, the outlook for patients with marked control deficits may seem bleak, but instead of focusing on potentially irreversibly damaged control capacities, one could also try to find a working point by focusing on relatively preserved cognitive functions. As previously noted, habits and automatisms seem to be rather unimpaired by alcohol abuse. So far, there has been comparatively little research on this therapeutic potential, but a few studies based on a retraining of automatic approach/avoidance tendencies towards alcohol-related stimuli have provided first hints for the efficacy of such interventions (Wiers et al., 2011; Naqvi and Morgenstern, 2015).

In this context, the probably best-known approach to altering unwanted AUD behavior via habits and automatisms (instead of addressing cognitive control), is cognitive bias modification (CBM) (for an overview, see Gladwin et al., 2017). Put simply, it is based on the aforementioned finding of S-R-driven alcohol consumption and the observation that untreated AUD patients show an automatic approach bias, which seems to be reduced in patients who benefit from AUD therapy (Gladwin et al., 2017). Based thereon, CBM aims to establish an automatic avoidance tendency towards alcohol-related stimuli, which is often done by asking patients to push a lever or joystick towards visual stimuli like pictures of soft drinks (or other non-alcohol stimuli) while pulling it away from alcohol-related stimuli (Wiers et al., 2011; Eberl et al., 2013; Boendermaker et al., 2016; Gladwin et al., 2017). In addition to this response-targeted CBM, the same research group has also investigated attentional bias modification (ABM) procedures aimed at reducing the amount of attention allocated to alcohol-related stimuli, but clinical effects of the latter still remain to be established. Response-based CBM has been shown to generalize to untrained visual stimuli (Wiers et al., 2011) and to reduce relapse rates in AUD patients without KS after 1 year (49.8% of *n* = 248 with CBM vs. 57.3% of *n* = 227 without) when used as an add-on to regular AUD therapy (Eberl et al., 2013).

But even with CBM, roughly half of the treated patients still experience relapses and not all studies using CBM are able to find a clear-cut beneficial effect on relapse rates (Wiers et al., 2015; Copersino, 2017). While a lack of motivation in some of the participants may have contributed to this (Gladwin et al., 2017), it is also conceivable that the reason for this lies in the specificity of the treatment as CBM targets only one specific aspect of habitual responding out of the wide range of S-R associations and the resulting addiction behavior in AUD.

This is why I would like to advocate for a well-known way of altering habits which is more comprehensive, but has so far not been applied to AUDs: the habit reversal therapy (HRT).

Habit reversal therapy encompasses several stages designed to alter dysfunctional habits without heavily relying on executive control and has already been proven to be effective in other disorders characterized by unwanted automatisms/habitual behavior, such as Tourette syndrome and chronic tic disorder or trichotillimania (Snorrason et al., 2015; Whittington et al., 2016; Yang et al., 2016).

During the initial “awareness training,” the patient is made aware of his/her habits and automatisms. In the case of AUD patients, this should probably include several aspects of their S-R association-based habitual alcohol consumption. To my mind, this should include identifying stimuli and situations triggering addictive behavior and also put a major focus on the different (chains of) responses constituting the habit.

In a subsequent therapy phase (“development of a competing response”), an effective competing habit or response, which needs to be carried out every time the urge to perform the initial unwanted habit/automatism emerges, is developed (in case specific behavior-eliciting stimuli have been identified during the initial phase, this would, however, also apply to situations where such stimuli are encountered – irrespective of whether they elicit craving). In this phase, the unwanted automatic behavior becomes altered or replaced by another habit/automatism which is established as part of the therapy. This is crucial as the approach requires only little cognitive control to effectively alter behavior in the long run. Importantly, this means that any irreversible cognitive control deficits that may have resulted from former alcohol abuse are not as much of an impediment to the success of this therapeutic intervention as it would have been in many alternative therapeutic approaches. In case of less complex unwanted automatisms such as tics, patients are often trained to develop a habit of performing a counteractive motor movement and the aforementioned CBM training already does something closely related by trying to counteract automatic alcohol approach tendencies by establishing competing avoidance responses. However, it has been recognized as a problem for standardized retraining strategies such as CBM that the range of S-R associations and implicit responses in AUD is far beyond the scope of this approach/avoidance aspect and substantially increases over the course of addiction (Copersino, 2017). In case the individual responses culminating in harmful alcohol consumption are carefully analyzed and dissected during the initial “awareness training” phase, it should, however, become possible to develop specific, individually tailored counteractive habits to several of these responses (like routinely doing sport after every frustrating event or day, screwing the lid of a bottle shut instead of opening it, or pouring alcohol into the sink instead of into a glass, just to make up a few examples^5^). The establishment of competing responses is further promoted by the therapy building block “generalization of new skills” that helps to generalize the competing response to as many relevant contexts as possible/necessary (which is something has mostly been neglected in previous therapeutic approaches). As a consequence, conscious and effortful controls are required less and less over time. Lastly, the block of “building motivation” is designed to motivate the patients to keep up with therapy (again without requiring too much top-down behavioral control). This aspect of HRT is important to maintain the patients’ compliance as the development and generalization of competing responses takes time and therefore does not yield immediate effects/rewards. Also, adequate motivation as well as the development of a positive long-term perspective seem to be a crucial prerequisite to yield positive outcomes when trying to manipulate automatic processes in AUD (Gladwin et al., 2017).^6^

While the development of competing habits/responses would certainly require an individually tailored approach for each patient, it holds the potential of breaking chains of responses that would otherwise culminate in alcohol consumption. Based thereon, HRT (probably also in combination with CBM) might provide an exciting new therapy option for AUD patients with severe and/or permanent executive control deficits who cannot sufficiently benefit from cognitive control training.

## Conclusion

Since HRT has not yet been applied and evaluated in the context of alcohol addiction, more research is needed to establish whether it provides an effective addition to or even replacement of control-focused AUD therapy. Also, we need to put further consideration into how potent competing responses can be developed. Importantly, this perspective does in no way intend to disregard the fact that the imbalance between habitual and goal-directed behavior is by far not the only mechanism at work in AUD, or that pharmacological interventions may provide valuable support for therapeutic advances. Yet, accumulating evidence strongly suggests that the alcohol-induced imbalance between goal-directed and habitual behavior may play a key role in staying abstinent. Hence, focusing on intact habitual/automatic mechanisms in addition to or maybe even instead of deficient cognitive control might equip us with a more effective tool to battle the current alcohol abuse and addiction epidemic, especially with respect to more severely impacted patients who likely suffer from permanent alcohol-induced brain damage. Against this background, I would like to advocate the application and scientific evaluation of HRT or similar therapies for alcohol abuse and addiction.

## Author Contributions

The author confirms being the sole contributor of this work and approved it for publication.

## Conflict of Interest Statement

The author declares that the research was conducted in the absence of any commercial or financial relationships that could be construed as a potential conflict of interest.

## Footnotes

1 http://www.who.int/gho/alcohol/consumption_levels/adult_recorded_percapita/en/

2 For example, Jason et al. (2006) reported that 65% of *n* = 75 alcohol addicts receiving “usual after-care" relapsed within 24 months after detoxification. Picci et al. (2014) found that 47% of *n* = 168 alcohol-dependent patients had already relapsed 6 months after their detoxification in a hospital. Kolla et al. (2015) reported relapse rates of 48% in patients undergoing treatment and 66% ITT in alcohol-dependent patients within 12 months after treatment (*n* = 119). Nalpas et al. (2003) reported that the mean time until the first relapse in *n* = 267 patients addicted to alcohol was between 3.37 and 5.89 months in four different treatment centers. As relapse rates are cumulative measures, those numbers rise even further when looking at longer follow-up time spans. For example, Garbusow et al. (2014) stated in their review that “on the average, 8 or 9 out of 10 alcohol-dependent patients relapse after detoxification,” but they did not provide a time span.

3 The underlying mechanisms can be explains within the framework of several dual-process theories. For example, the associative cybernetic model by de Wit and Dickinson (2009) suggests a mechanistic account of how alcohol abuse may lead to behavioral autonomy based on changes in outcome-response associations and response-outcome associations. In contrast to this, the Rł model suggests a continuum of automatic versus reflective processing where response selection depends on how much evaluation precedes selection (Gladwin et al., 2011, 2017).

4 Importantly, the imbalance of habitual and goal-directed control behavior is not only present in AUD patients (Sebold et al., 2014), but can also be found in animal models (Corbit and Janak, 2016) and healthy humans during high-dose alcohol intoxication (Stock et al., 2014, 2016), which suggests that alcohol is a causal factor for this imbalance.

5 These examples are just meant to provide a rough mechanistic idea as the development of effective competing responses likely requires an individually tailored approach (which cannot be illustrated in detail without a case study) and extensive therapeutic experience.

6 One of the reasons for this could be that non-conscious cognitive and motivational processes are responsible for the effects of mental contrasting, which may produce either active goal pursuit or active goal disengagement, depending on the expectation of success (Oettingen, 2012).
